# Book Reviews

**Published:** 1915-06

**Authors:** 


### BOOK REVIEWS

Books mentioned may be inspected at and ordered through this office. So far as possible, books received in any month will be reviewed in the issue of the second month following.

**A Practical Treatise on Diseases of the Skin.** By Oliver S. Ormsby, M. D., Professor of Skin and Venereal Diseases in the Rush Medical College, Chicago. Octavo, 1168 pages, with 303 engravings and 39 plates in colors and monochrome. Cloth, $6.00, net. Lea & Febiger, Publishers, Philadelphia and New York.

This is a large, systematic treatise, comparable to modern works on practice, surgery, and gynaecology. The first 135 pages are devoted to general considerations of histology and physiology, diagnosis, and therapeutics. Skin diseases are divided into the following classes: hyperaemias and inflammations, haemorrhages, hypertrophies, atrophies, pigment anomalies; new growths, neuroses and parasitic. 140 pages are devoted to the dermal appendages and a brief chapter discusses diseases of mucous membranes coming within the province of dermatology. From the standpoint of classification and nomenclature, the work approaches theoretic perfection, at the same time, no impossible effort has been made to make facts fit theories.

---

**Practice of Medicine.** By Walter Sands Mills, A. B., M. D., Professor of Medicine, New York Homoeopathic Medical College and Flower Hospital; Physician to the Metropolitan Hospital, and to the Tuberculosis Infirmary, Department of Public Charities. 705 pages. Buckram. Gilt top. $5.00, net. Philadelphia: Boericke & Tafel. 1915.

This is a thoroughly but modernly homoeopathic text book. Definitions, diagnostic points, etiology, historic notes, morbid anatomy and pathology are clearly and briefly stated, with headings in black type, under each disease. The author does not scorn mention of "old school" treatment. Unlike some of his confreres, the author approves of various biologic tests and vaccinations but avoids the extreme of optimism which has blemished the writing of many of the "old school." In fact, he clearly recognizes that such methods, instead of being antagonistic to homoeopathy are practical developments of its primary tenets. For instance, speaking of tuberculin he says "Now many authorities begin with very small doses, one-millionth to one-billionth of a milligram or less—the homoeopathic 6x to the 9x potency. . . . . Could anything approach more nearly the true homoeopathic method?" The work is strictly that of a clinician, familiar with the results of laboratory workers but not attempting to introduce into a work on practice, details more properly left to work on pathology, chemistry, and special technic. We do not need to recommend this work to our homoeopathic colleagues save to say that it is far in advance of many superficial works which have appeared and which were mere personal jottings of routine experience. But, while admitting a personal preference for certain standard works on practice, we feel that physicians generally should study this presentation of homoeopathy, if for no other reason, to have an actual basis for judgement of the present standards of a school of medicine no longer separated from them except as to certain conceptions of therapeutics and never at variance with general profession standards and aims.

---

**Lectures on the Heart.** By Thomas Lewis, M. D., F. R. C. P., D. Sc., London. Published by Paul B. Hoeber, N. Y. 124 pages, 83 illustrations, $2.00.

These lectures comprise the one before the Harvey Society of N. Y., the three of the Herter foundation, Baltimore, and one before the faculty of medicine of McGill, Montreal, in the autumn of 1914. They are based mainly on experimental and clinical work with the electro-cardiograph. General principles and clinical applications of this method are thoroughly discussed. On the one hand, the author has, so far as possible, confined himself to essentially modern research on the cardiac and circulatory phenomena; on the other, he has brought to bear on the subject a wealth of experience derived from older methods. His descriptions are lucid and his conclusions conservative.

---

**Swat the Fly.** A one act fantasy by Eleanor Gates, Arrow Publishing Co., 116 West 59th, N. Y. 25 cents.

Animal experimentation, bacteriology and sanitation are dramatized, by a doctor, an anti-vivisectionist, a fly, a horse, a dog and a cat in a way that could hardly be anticipated by medical men, who are apt to regard such matters in a hard-headed way. The lay reader will, perhaps derive a somewhat fantastic and exaggerated view of sanitary principles but the general moral seems to be good.

---

**Federal Narcotic Record Book,** 25 cents, Abbott Alkaloidal Co., Chicago.

This is a small book, containing the Law in full, a list of the Abbott preparations containing narcotics, with dose in each tablet, and blanks for inventory and record of narcotics dispensed or distributed. It is a cheap and convenient guide for those embarrassed by this new legal restriction.

---

**A Reference Book on the Federal Narcotic Law,** for physicians, druggists, dentists and veterinarians, by Albert Dean Currier of the Chicago Bar and Daniel R. Forbes, late with U. S. Board of Food and Drug Inspection. 25 cents.

This contains the Harrison Law, comments upon it under various headings, gives a synopsis of rulings and a list of commonly used drugs, officinal and proprietary, affected by the law. The book is indexed but contains no blanks for record.

---

**Medical Ethnology.** By Chas. E. Woodruff, A. M., M. D., Lt. Col. U. S. A., retired. 320 pages, $2.00. Rebman Co., 141-145 West 36th, N. Y.

This contains observations on evolution of man, the development of physical characteristics, especially pigmentation under action of the sun, the causes of extinction of migrants and psychology as influenced by race. The last chapter deals with applications of ethnology in medicine. It is a work which deserves careful study, as it is exceedingly difficult to epitomize the conclusions or to draw general rules without qualification.

---

**The Practical Medicine Series.** Vol. 9, Series of 1914, Skin and Venereal Diseases, edited by W. L. Baum and J. H. Mitchell; Miscellaneous Topics, edited by Harold N. Moyer. $1.35 separately.

**Vol. 1, Series of 1915, General Medicine,** edited by Frank Billings and J. H. Salisbury. $1.50 separately.

This series is published by the Year Book Co. of Chicago, each annual series consisting of ten volumes and being distinguished by different colored bindings. The price of an annual series is $10. The volumes appear at intervals of about a month. This is the most practical review of current medical literature published.

---

**Surgery of the Blood Vessels.** By J. Shelton Horsley, M. D., Richmond. Published by the C. V. Mosby Co., St. Louis. 304 pages, 89 illustrations. $4.

After briefly discussing the histology of the vessels, the indications for suturing and the history of blood vessel surgery, the technic of suturing and anastomosis and reversal of current are considered. Transfusion, haemorrhage, thrombosis and embolism, occlusion of the mesentric vessels, aneurysms, tumors of the blood vessels, varices of various kinds follow and a special chapter is devoted to transplantation of the anterior temporal artery. In few branches of medicine have such signal practical advances been made as in this field and a monograph embracing modern surgery of the vessels fills an actual want. The author is thoroughly familiar with his subject and the work, even mechanically in letter press and illustrations is of the highest order.

---

**Infection and Immunity.** A text book of Immunology and Serology. For Students and Practitioners. By Charles E. Simon, B. A., M. D., Professor of Clinical Pathology and Experimental Medicine, College of Physicians and Surgeons, Baltimore; Pathologist to the Union Protestant Infirmary, the Women's Hospital of Maryland and the Mercy Hospital, Baltimore. Third edition, enlarged and thoroughly revised. Octavo, 351 pages, illustrated. Cloth, $3.25 net. Lea & Febiger, Publishers, Philadelphia and New York, 1915.

The rapid succession of editions indicates the deserved popularity of this work. Considerable qualifications have been made to the Wassermann test, the prevention of anaphylactic shock has reached a practical stage, Schisk's method of noting whether a sufficient quantity of diphtheria anti-toxin is present has introduced the matter of dosage into this protective measure, improvement has been made in the serum treatment of tetanus and Hodgkin's disease has come into line for vaccine treatment. These highly important advances, in addition to the opportunities always presented for improving a new edition in minor details, have created a demand for a third edition, quite aside from the exhaustion of the second edition. The book is one which the general practitioner should use, even if he does not attempt the technical details of amynology. Immediate recognition of indications by the man first seeing the case of infectious disease or otherwise amenable to diagnosis or treatment by biologic methods, is not merely a matter of scientific education but of life-saving importance.

---

**Transactions of the American Gastro-Enterologic Association,** 17th Annual Session, Atlantic City, June 22 and 23, 1914.

This contains a list of officers and members, constitution and by-laws and reprints of the papers read at the session.

---

**Atlas of the Differential Diagnosis of the Diseases of the Nervous System.** By Dr. Henry Hun, Albany. Published by the Southworth Co., Troy. 287 pages, illustrated.

This is the second, revised and extended edition of a work which we reviewed last year. It is not only unique as a work on nervous diseases, but as applying to medicine to more than a superficial degree, the plan of analysis long familiar to botanists, conchologists and other biologists and even, in its general principles, to genealogists. The table of contents is an index of indexes of the book itself. The various tables stimulate thorough investigation of the cases arising in practice. If they went no farther than this, they would lead to a more careful routine of examination and would avoid oversight of important diagnostic details. But, each symptom noted and each physical test made is traced by appropriate tables, quite readily found by the index, as far as possible toward an accurate diagnosis. Combining the results for individual diagnostic points, the ultimate diagnosis is reached with almost unerring accuracy. We do not doubt but that individual cases will occur in which the logical path to an ultimate formal diagnosis cannot be traced—at least, this would be our experience with regard to the line of work with which we are more familiar—still, in the great majority of instances, the diagnosis can be reached by the analysis. Moreover, the analytic tables are more than mere systems of applying disease terms. They include statements as to the general nature of processes causing symptoms and hence are diagnostic in the true as well as the superficial sense.

---

**Materia Medica and Therapeutics.** A text book for Nurses. By Linette A. Parker, B. S., R. N., New York. Published by Lea & Febiger, Philadelphia and New York. 311 pages, 29 engravings, 3 plates. $1.75.

This is one of the blue, Nurses' Text Book series. The initial chapters are devoted to weights and measures, strength of solution, classification of Galenicals, etc. The regular part of the book deals with drugs, the more important ones being discussed in some detail while a list is given of drugs of minor importance, including some which individuals would consider more seriously. Prescription writing is presented briefly. A chapter on legislation concerning drugs is a wise inclusion. Psychotherapy, electricity, X-Rays, heliotherapy, serums and vaccines are well discussed. The work is well written and, indeed, shows plainly the influence of a liberal education, professional experience and experience in didactics.

---

**The Clinics of John B. Murphy,** April, 1915, published by the Saunders Co., Philadelphia.

It is of the usual high character. Dr. Murphy's insistence upon the necessity of the early recognition of acute osteomyelitis by the physician in general practice is a most forcible object lesson. It is to be hoped that he will not tire of driving this lesson home, for the profession seems to be particularly remiss in this disease. It is most easily recognized in the first onset so there is no possible excuse for failure to secure surgical treatment at a time when the results of that treatment are brilliant. Delay surely produces necrosis of bone with its deplorable course so well known to all of us. The usual bone cases are contained in this volume. We hope that Dr. Murphy will be induced to incorporate the principles and technic of his bone and joint work into a monograph in order to place it before the profession in a comprehensive and compact form.—C. W. H.

---

**Oxygen Injected in Tetanus.** Leger, Compt. rendues, Soc. de Biol., 1915, p. 3, connects a hypodermic needle to a balloon of oxygen and injects as much as can be introduced without producing gaseous embolism. Recovery occurred in three cases, two apparently hopeless.

